# Leveraging Artificial Intelligence for Digital Symptom Management in Oncology: The Development of CRCWeb

**DOI:** 10.2196/68516

**Published:** 2025-06-16

**Authors:** Darren Liu, Yufen Lin, Runze Yan, Zhiyuan Wang, Delgersuren Bold, Xiao Hu

**Affiliations:** 1 Department of Computer Science, Laney Graduate School, Emory University, Atlanta, GA, United States; 2 Nell Hodgson Woodruff School of Nursing, Emory University, 1520 Clifton Rd NE, Atlanta, GA, 30322, United States, 1 3104986136; 3 Center for Data Science, Emory University, Atlanta, GA, United States; 4 Winship Cancer Institute, Emory University, Atlanta, GA, United States; 5 Department of Systems and Information Engineering, University of Virginia, Charlottesville, VA, United States, 1 3104986136; 6 The Wallace H. Coulter Department of Biomedical Engineering, Georgia Institute of Technology, Atlanta, GA, United States

**Keywords:** colorectal cancer, health disparity, health equity, generative artificial intelligence, large language model, software engineering, artificial intelligence

## Abstract

Digital health interventions offer promise for scalable and accessible health care, but access is still limited by some participatory challenges, especially for disadvantaged families facing limited health literacy, language barriers, low income, or living in marginalized areas. These issues are particularly pronounced for patients with colorectal cancer (CRC), who often experience distressing symptoms and struggle with educational materials due to complex jargon, fatigue, or reading level mismatches. To address these issues, we developed and assessed the feasibility of a digital health platform, CRCWeb, to improve the accessibility of educational resources on symptom management for disadvantaged patients with CRC and their caregivers facing limited health literacy or low income. CRCWeb was developed through a stakeholder-centered participatory design approach. Two-phase semistructured interviews with patients, caregivers, and oncology experts informed the iterative design process. From the interviews, we developed the following 5 key design principles: user-friendly navigation, multimedia integration, concise and clear content, enhanced accessibility for individuals with vision and reading disabilities, and scalability for future content expansion. Initial feedback from iterative stakeholder engagements confirmed high user satisfaction, with participants rating CRCWeb an average of 3.98 out of 5 on the postintervention survey. Additionally, using generative artificial intelligence tools, including large language models like ChatGPT and multimedia generation tools such as Pictory, complex health care guidelines were transformed into concise, easily comprehensible multimedia content, and made accessible through CRCWeb. User engagement was notably higher among disadvantaged participants with limited health literacy or low income, who logged into the platform 2.52 times more frequently than nondisadvantaged participants. The structured development approach of CRCWeb demonstrates that generative artificial intelligence–powered multimedia interventions can effectively address health care accessibility barriers faced by disadvantaged patients with CRC and caregivers with limited health literacy or low income. This structured approach highlights how digital innovations can enhance health care.

## Introduction

Colorectal cancer (CRC) is the third most common cancer and the second leading cause of cancer-related deaths in the United States [1]. For patients with CRC, educational materials play a critical role in understanding their diagnosis, exploring treatment options, self-managing their side effects and symptoms, and navigating posttreatment care, ultimately empowering them to make informed decisions about their health [2-4]. However, most of these materials are primarily text-based, which poses significant accessibility challenges for disadvantaged populations, such as individuals with low income or limited health literacy [5].

Caregivers, typically family members who provide emotional and physical support, frequently experience similar distressing symptoms, making it equally challenging for them to engage with complex medical content [6,7]. For disadvantaged populations with limited health literacy or low income, these barriers are even more pronounced, which reduces their ability to access and understand crucial health information [8]. Many educational materials are only available in English, further excluding non-English-speaking individuals from receiving critical guidance on symptom management and supportive care [9]. These factors combine to create a critical gap in the ability of patients with CRC and their caregivers to effectively manage symptoms and make informed decisions about their treatment, highlighting the urgent need for more accessible solutions.

Recent advancements in generative artificial intelligence (GenAI) offer a transformative solution to these challenges. GenAI can convert traditional text-based educational materials into multimedia formats at a fraction of the cost and time, making it an efficient and scalable option [10,11]. In this work, we introduce CRCWeb, a novel GenAI-driven digital health mobile platform designed to provide accessible, tailored symptom management resources to patients with CRC and their caregivers. Powered by state-of-the-art GenAI models like ChatGPT [12], CRCWeb transforms dense health care texts into digestible multimedia formats, including videos and audio, concise and easy-to-understand health knowledge, practical activity prompts, and quizzes. This approach makes essential health knowledge on symptom management and cancer care more accessible to individuals with CRC facing low literacy and other accessibility challenges [13]. By reducing the cognitive load required to process health information, CRCWeb empowers patients and caregivers with easy-to-understand materials, improving their abilities to manage symptoms and adhere to treatment plans and guidelines. Therefore, the purpose of this viewpoint is to describe the development of CRCWeb, which leverages GenAI for digital symptom management in patients with CRC and their caregivers, and present preliminary data to support its feasibility and potential to reduce barriers to accessing health information and improve user engagement, satisfaction, and symptom management.

## Contextual Background

We used a stakeholder-centered participatory design approach to develop CRCWeb at the Nell Hodgson Woodruff School of Nursing, Emory University, Atlanta, Georgia, United States. The motivation for this project was to develop and test a symptom management tool tailored to the needs of disadvantaged patients with cancer, particularly those with limited health literacy or low income. The project was led by a multidisciplinary research team with expertise in oncology, nursing, AI, technology, psychology, behavioral science, and clinical trial design. The development and evaluation of CRCWeb took place between November 2022 and May 2025.

## Designing CRCWeb

### Overview

CRCWeb was developed through an iterative stakeholder-centered participatory design approach [14,15]. We conducted 2-phase, semistructured interviews using an interview guide developed from the existing literature that included open-ended questions and probes to elicit their specific needs, challenges, and expectations for a technology-based intervention tool for symptom management during chemotherapy treatment. Each interview lasted between 30 and 45 minutes and was conducted in either a private conference room at the clinic or virtually via Zoom (Zoom Video Communications, Inc), depending on the participant’s preference. All interviews were audio-recorded and transcribed, and field notes were taken to document nonverbal cues. Content analysis was used to analyze the qualitative data through Dedoose (SocioCultural Research Consultants) and was completed in 4 steps: data preparation, writing memos, coding, and categorizing and connecting [16].

### Semistructured Interviews: Phase 1

In Phase I, 11 patients with CRC, 8 caregivers, and 4 oncologists were asked about their perspectives and suggestions for a technology-based intervention to manage symptoms. We gathered their feedback before the platform’s development. This early involvement of key users allowed us to ensure that the platform would be designed to address their specific needs, challenges, and expectations from the outset. During the interview, the participants were guided by the questions in Table 1. We started by discussing their needs for a technology-based intervention tool during chemotherapy (Question 1) and their experiences with existing technological tools for symptom management (Question 2), allowing us to identify key gaps in current digital solutions. Providers were also asked to offer suggestions for the development of this tool (Question 3).

**Table 1.** Two-phase semistructured interview questions designed for iterative stakeholder-centered participatory design^a^.

Phase	Examples of interview questions
I	1. Can you describe your need for a technology-based intervention tool to manage symptoms during chemotherapy? 2. Have you used any technological tools for symptom management? If so, which ones have you used, and what is your experience with them? 3. Do you have any suggestions on developing a technology-based intervention program for patients and caregivers? What are they?
II	4. What are your likes and dislikes regarding the CRCWeb intervention components (eg, family involvement, symptom management, and coping strategies), delivery methods (eg, doses and intervals), and formats (eg, video, audio, and evaluations)? 5. How easy or difficult was it for you to navigate and understand this technology tool? 6. What challenges or barriers have you encountered when accessing cancer care and symptom management? 7. In your opinion, how can we achieve health equity in cancer care? 8. What are the facilitators and challenges related to implementing a technology-based intervention for patients and caregivers?

a Questions 1 to 3 were used in phase I to collect participants’ needs for CRCWeb prior to development, while questions 4 to 8 were used in phase II to iteratively gather feedback from participants to propose new designs and functionalities.

### Semistructured Interviews: Phase 2

In Phase II, we expanded the participant pool beyond the original 23 from Phase I to include a more diverse group: 5 additional patients and 5 caregivers, as well as a palliative care physician, a nurse practitioner, and a clinical leader. These participants were iteratively asked to provide feedback on CRCWeb’s proposed content, including family involvement, symptom management, and coping strategies, as well as the delivery methods and formats (Question 4) and their user experiences (Question 5). Additionally, the interviews addressed topics such as barriers to accessing cancer care and symptom management (Question 6), participants’ perspectives on achieving health equity (Question 7), and the facilitators and challenges associated with implementing the intervention (Question 8). After each iteration, we proposed new designs and functionalities based on participants’ feedback. Given that over half of the participants that we interviewed came from disadvantaged backgrounds with limited health literacy or low income and were unfamiliar with technology, the iterative design process allowed them to actively engage with CRCWeb during its early development stages, ensuring that the approach aligned with their needs and preferences. These participants provided valuable insights into the specific needs that CRCWeb aimed to address, particularly the need for improved accessibility to health care resources and information.

## Ethical Considerations

The study protocol (STUDY00004750) was approved by the institutional review board at Emory University. Written consent was obtained from participants. All participants were informed of the voluntary nature of their participation and their right to withdraw at any time without consequence. All research data were anonymized to maintain confidentiality. Study materials were securely stored and accessible only to authorized research team members. Participants received up to $60 compensation for their involvement. The study was conducted in accordance with the US Common Rule (45 CFR 46).

## Design Principles

The design principles outlined in Table 2 were identified from our 2-phase semistructured interview transcripts using content analysis. To reduce the learning curve of using a new app, particularly for disadvantaged populations with limited health literacy or low income, we applied design principle 1, ensuring that CRCWeb includes intuitive and user-friendly navigation features. To increase participant engagement, we implemented design principle 2 to include multimedia components to enrich the content [17,18]. Design principle 3 ensures that the learning modules are concise, containing only essential information. This design principle minimizes learning time and makes the content easier for disadvantaged individuals with limited health literacy to comprehend. To assist individuals with vision impairments and reading disabilities, we designed the vision principle, optimizing CRCWeb to enhance accessibility for these users. Design principle 5 was implemented to ensure that the system is scalable, allowing for the inclusion of additional educational materials in various formats in the future without requiring changes to the system architecture. These qualitative data are invaluable in identifying key features that would drive the development of CRCWeb.

**Table 2.** Five design principles were outlined from the 2-phase semistructured interviews with patients and caregivers^a^.

Design principle	Explanation
User-friendly principle	Our platform should feature intuitive and user-friendly navigation.
Multimedia principle	Our platform should feature multimedia components.
Concise principle	Extraneous material should be excluded to keep the content short and easy to understand.
Vision principle	Our app should feature functions that help people with limited vision to access educational content.
Scalability principle	Our platform should be scalable to include more topics and content formats in the future.

a These open-ended questions aim to address accessibility challenges for disadvantaged populations facing limited health literacy or low income by incorporating intuitive navigation, multimedia components, concise and easy-to-understand content, and functionalities designed to assist individuals with limited vision.

## Development of CRCWeb

**Figure 1.**
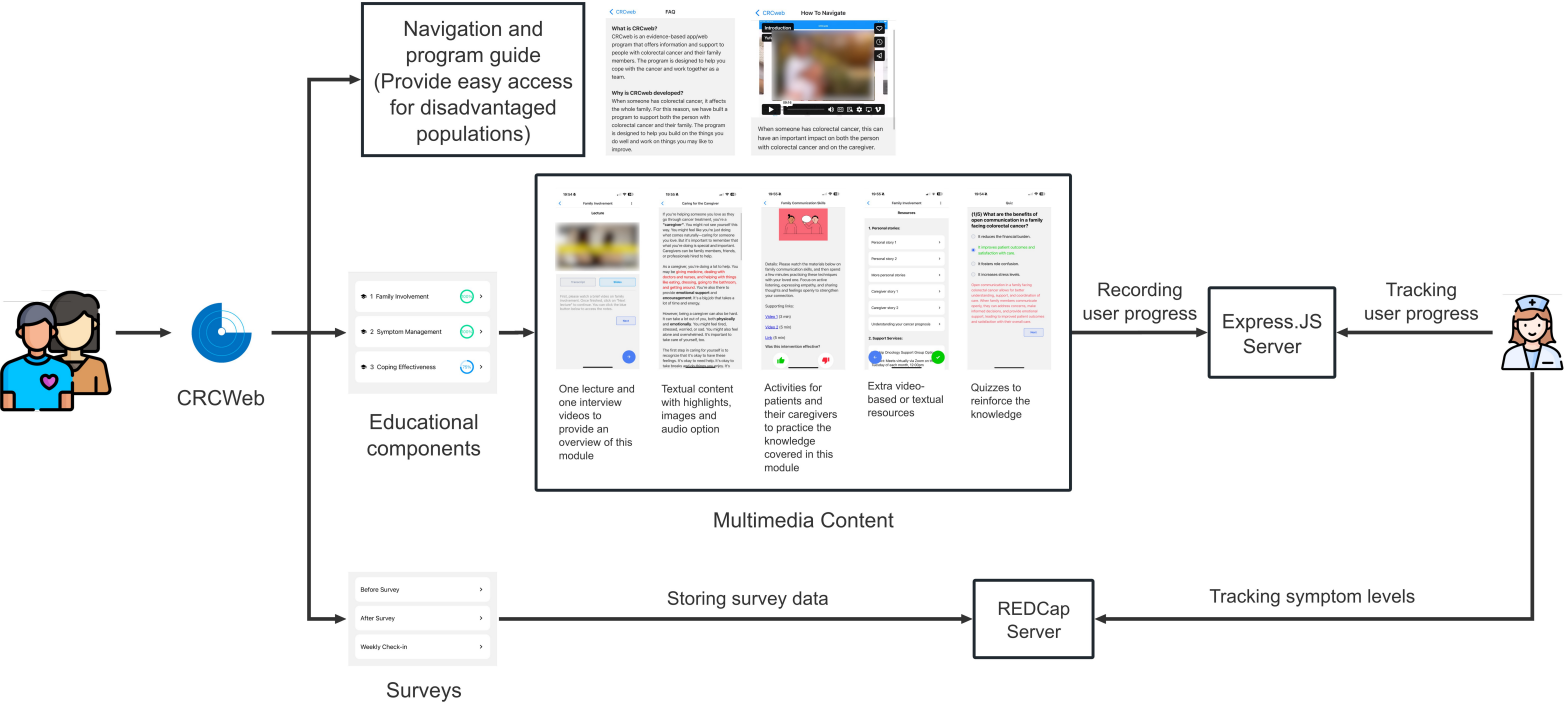
System architecture of CRCWeb, highlighting its 3 core components: navigation and program guide, educational components, and surveys. The educational components feature 5 multimedia sections: lectures, content, activities, resources, and quizzes. User progress is securely stored on our server, ensuring confidentiality while enabling administrators to monitor and track advancement. REDCap: Research Electronic Data Capture.

The development of CRCWeb strictly followed the design principles outlined in the previous section. As shown in Figure 1, CRCWeb contains 3 main components: navigation and program guide, educational materials, and surveys. The navigation and program guide simplify access while the educational components offer engaging, multimedia content tailored to different learning needs. The survey feature tracks symptom levels and user progress, with data securely stored on the REDCap (Research Electronic Data Capture; Vanderbilt University) server. Then, we discussed in detail how CRCWeb’s design increased accessibility by focusing on these 3 key components.

## Functionalities of CRCWeb

**Figure 2.**
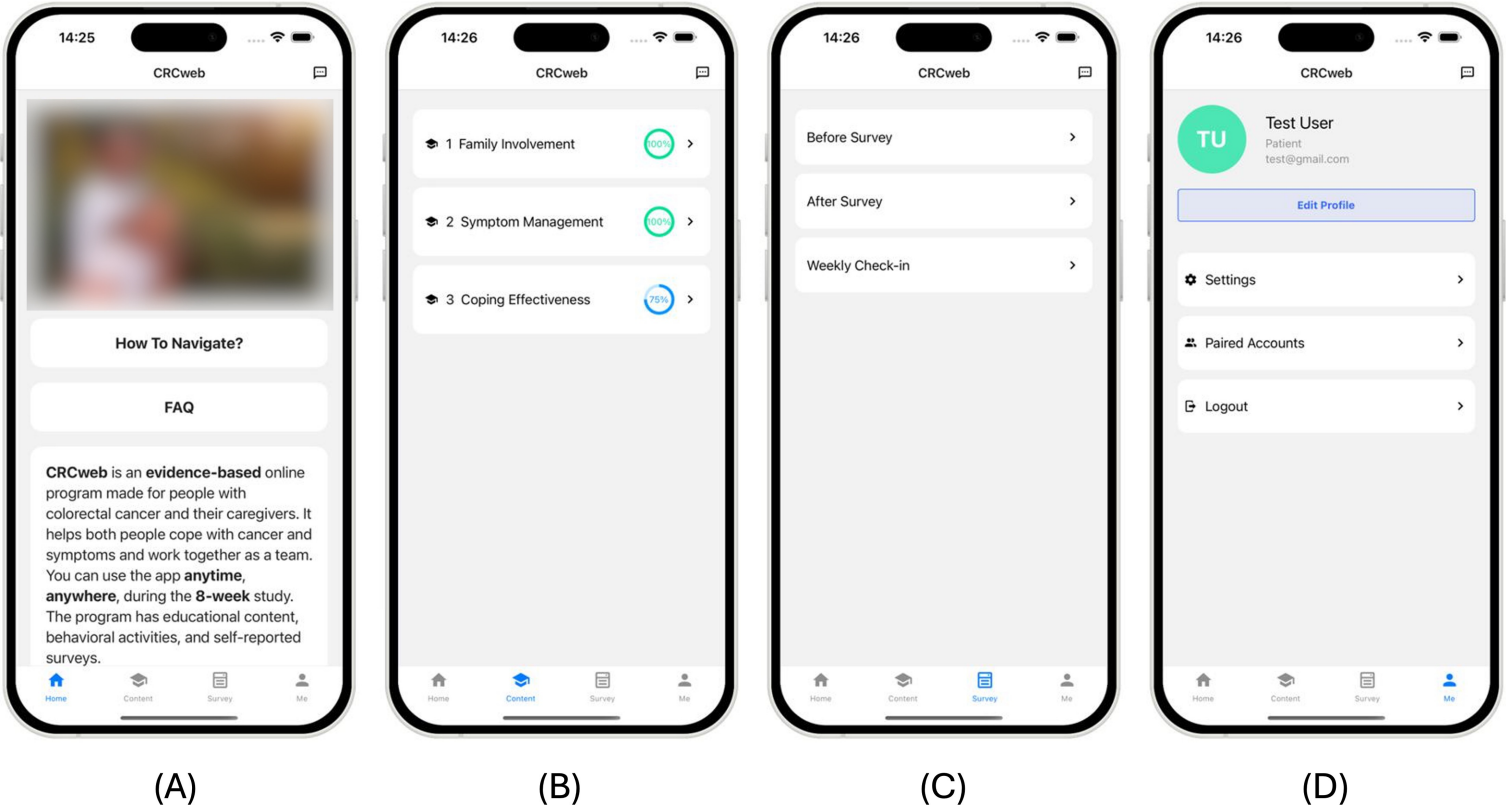
System architecture of CRCWeb, highlighting its 3 core components: navigation and program guide, educational components, and surveys. The educational components feature 5 multimedia sections: lectures, content, activities, resources, and quizzes. User progress is securely stored on our server, ensuring confidentiality while enabling administrators to monitor and track advancement.

**Figure 3.**
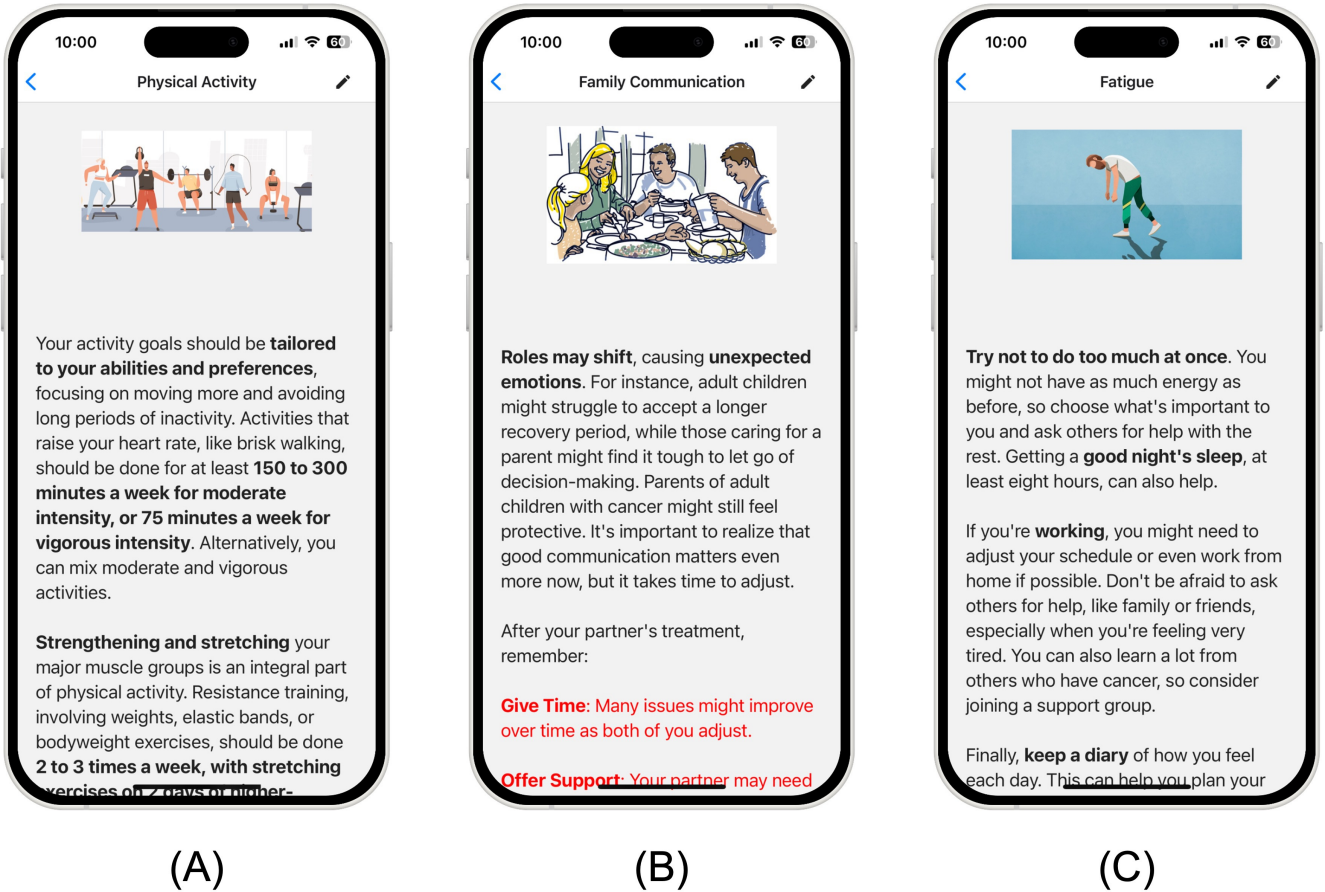
Examples of educational topics in CRCWeb: (A) Physical Activity, offering practical exercise recommendations; (B) Family Communication, guiding effective caregiver-patient discussions; and (C) Fatigue, providing practical strategies for managing energy levels during treatment.

As shown in Figure 2, CRCWeb’s main interface includes 4 navigation tabs at the bottom of the screen: Home, Content, Survey, and Account. This streamlined layout is crucial for enabling users to easily access the app’s core functionalities without confusion. By providing a consistent and simple navigation system, CRCWeb ensures that users, especially those with limited technological proficiency, can effortlessly switch between essential features, aligning with design principle 1. As shown in Figure 2A, users can also quickly navigate between sections using a button located in the top right corner. Additionally, the large blue buttons at the bottom of the screen, as displayed in Figure 2C, allow users to move to the next or previous sections within a module. When a user reaches the final section, the right button turns green, indicating that they have completed the current module. To further assist users in tracking their progress, as demonstrated in Figure 2B, a green check mark appears next to the title of a section when it is finished, providing a visual reminder of completion. The Home tab includes essential resources such as “How to Navigate?” and “FAQ” sections, designed to guide users through the platform and answer common questions. These features are critical for users who may not be familiar with technology, ensuring that everyone can easily access and use CRCWeb’s resources. Users can also send direct messages to the research team for additional support by tapping the message icon in the top right corner. This feature is particularly useful for disadvantaged users with limited access to advanced technologies who may require additional help to navigate the platform or understand the content. As shown Figure 3B, the Content tab grants users access to educational modules, complete with a progress bar that visually tracks their completion status. This progress bar is essential in keeping users motivated and aware of their learning journey. The Survey tab, illustrated in Figure 2C, enables users to complete pre- and postintervention surveys, as well as weekly symptom check-ins. This structure allows CRCWeb to deliver notifications that adjust the learning experience based on user progress and survey responses. Finally, the Account tab, shown in Figure 2D, enables users to manage personal information and settings and pair their account with a caregiver. This paired account feature fosters collaboration, enabling patients and caregivers to share learning progress and better coordinate care efforts.

**Figure 4.**
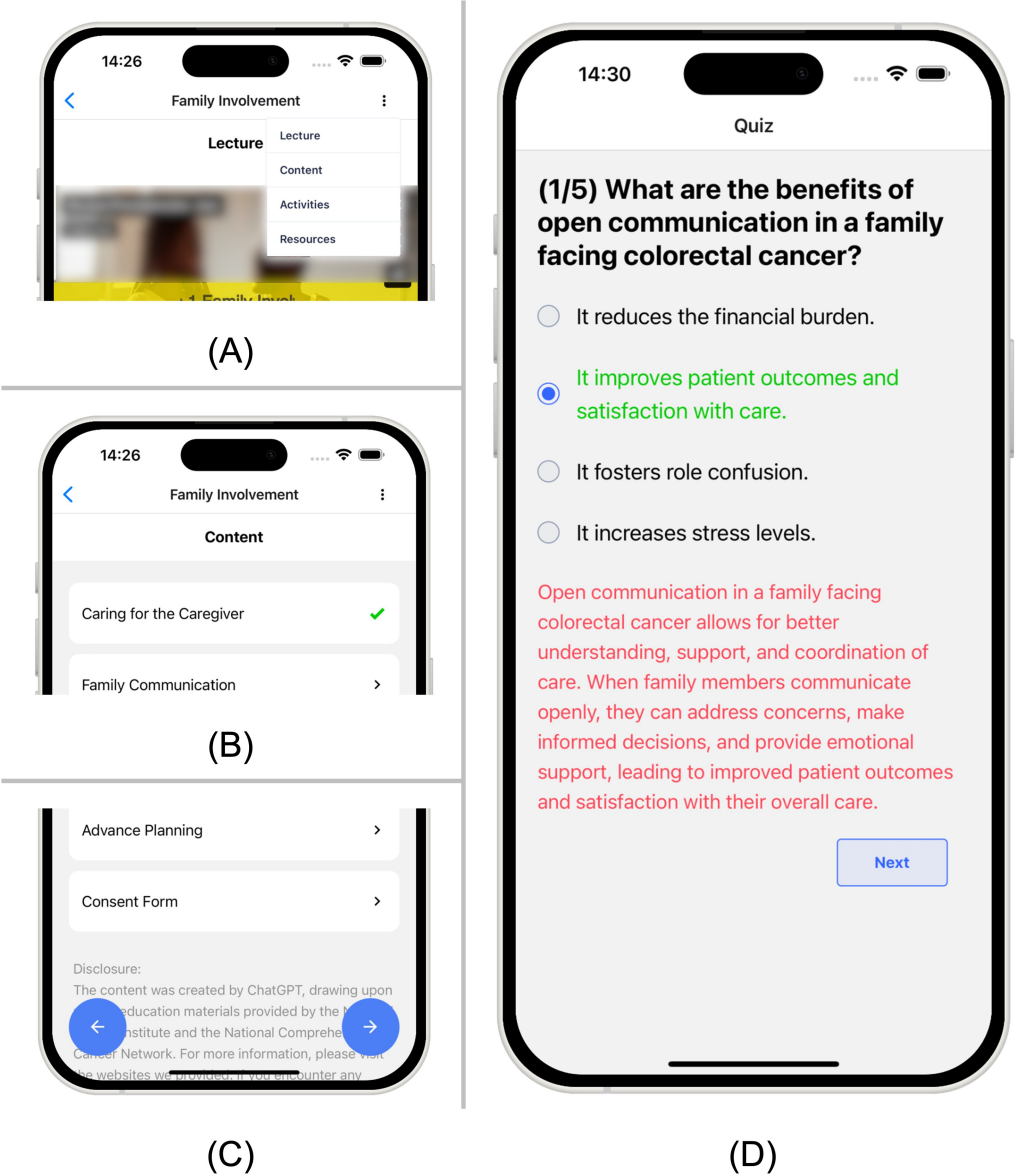
The user-friendly navigation features of CRCWeb: (A) a drop-down menu to quickly navigate through different sections; (B) a green check mark to indicate when a section has been reviewed; (C) large buttons for navigating between sections; and (D) quizzes with immediate feedback to inform users if they answer correctly and providing a detailed explanation.

One of the key tools supporting this interactivity is the smart content tagging system, which enhances the readability and accessibility of text-based materials. The content tagging system allows critical information to be highlighted using different font weights, colors, and multimedia elements. For example, key points can be bolded or marked in red or blue to guide users’ attention, as shown in Figure 3. In addition, users can choose to increase font size in settings. Driven by design principles 2 and 4, this system ensures that important information is easy to spot, particularly for users with low literacy or cognitive challenges. Additionally, for users with reading difficulties or visual impairments, we integrated a text-to-speech option that allows users to listen to the content instead of reading it. This feature is available throughout the app, ensuring that all users—regardless of their abilities—can engage with the educational materials. The system is designed to strip out tags before converting the text to speech, preventing unnecessary audio distractions. To reinforce learning, each module concludes with a quiz, as shown in Figure 4D. The quizzes provide immediate feedback, informing users whether their answers are correct, followed by explanations to deepen their understanding. Users are encouraged to achieve at least 80% accuracy before moving on to the next module. Notifications are sent to remind users to retake quizzes if they do not meet this threshold, ensuring that learning is reinforced and that users fully comprehend the material before progressing.

To ensure CRCWeb can be accessed on a wide variety of devices, we built the platform using the React Native framework [19]. This allows the platform to be deployed as an iOS app, Android app, and web app from a single code base, ensuring maximum accessibility across platforms. Since most disadvantaged populations with limited health literacy or low income are using older versions of Android devices, we made additional efforts to optimize CRCWeb’s performance in the Android environment to ensure a smooth, bug-free experience. Following an agile software development process [20], we continuously refined the platform based on feedback from internal testers and semistructured interviews with patients and caregivers, resulting in an iterative and user-driven design. The backend server of CRCWeb is built using the Express.js framework [21] and object-relational mapping [22] to accommodate any relational database, allowing flexibility in terms of database management while ensuring robust performance and scalability.

## Educational Content

### Tailoring Educational Materials for Symptom Management Using GenAI

CRCWeb’s educational content is organized around 3 core modules—Family Involvement, Symptom Management, and Coping Effectiveness—all of which are informed by Stress Coping Theory [23] and Family Systems Theory [24]. These theories emphasize the critical role that psychological resilience and family dynamics play in how individuals manage chronic conditions like CRC. Specifically, Stress Coping Theory highlights how emotional and cognitive responses influence symptom management, while Family Systems Theory indicates the importance of involving family members in the care process. By integrating these theoretical frameworks, CRCWeb was designed to improve both patient and caregiver outcomes and engagement, particularly for disadvantaged populations with limited health literacy or low income. Therefore, we hypothesized that enhanced family involvement and targeted psychosocial education alleviated the symptom burden for patients with CRC and their caregivers. This tool aimed to provide the knowledge, strategies, and emotional support necessary to manage symptoms effectively, ultimately improving both the patients’ and caregivers’ quality of life.

Each module is structured into 5 key sections: lectures, content, activities, resources, and quizzes. As illustrated in Figure 1, the educational materials were tailored into 4 distinct formats and distributed across different sections of CRCWeb: videos in the lectures section, textual content in the content section, practical activities in the activity section, extra-textual and video-based resources in the resources section, and quizzes in the quiz section. These educational materials were developed in alignment with guidelines from the National Cancer Institute [25] and the National Comprehensive Cancer Network [26], ensuring that the content is evidence-based and authoritative. As shown in Figure 3, the topics address a comprehensive range of practical, emotional, and physical challenges faced by patients with CRC and their caregivers. These topics are crafted to not only educate but also empower patients and caregivers, encouraging them to take an active role in managing cancer treatment and improving overall well-being.

To improve both engagement and accessibility, we integrated multimedia content throughout all modules, in alignment with design principle 2. Drawing from the semistructured interviews, we developed 5 distinct types of content—videos, slides, textual content, practical activities, extra resources, and quizzes—tailored to fit within the 5 sections of the program. Each lecture section includes 2 short videos: a primary lecture video that provides a comprehensive overview of the module’s content and a supplemental video featuring interviews with patients with CRC who share their lived experiences. These videos are complemented by slides that offer additional visual summaries, making the information accessible to users with different learning preferences. Each content section features concise textual information, complemented by images and highlighted key points. The activity section offers practical activities with detailed instructions, while the resources section provides additional more detailed resources. The quiz section at the end of each module includes 5 quizzes to reinforce learning.

In the activities section, users engage in 9 digital exercises (3 activities per module) designed to reinforce what they learned in the lectures and content sections. These activities are tailored to daily life routines, such as symptom tracking and communication exercises between patients and caregivers. The activities also include a rating feature that allows users to provide feedback on their experience through thumbs-up or down ratings and optional comments. This feedback loop is a crucial part of CRCWeb’s design, as it enables continuous refinement of activities based on real-time user input, ensuring that the content remains relevant and effective.

The resources section offers optional supplementary materials for users who wish to delve deeper into specific topics. These include additional videos, articles, and external links to trusted cancer resources. In response to user feedback, we curated this section to ensure that it provides meaningful yet nonoverwhelming options for further exploration. For example, users can access interviews with others managing similar symptoms, providing both practical tips and emotional support through shared experiences.

Each module concludes with a 5-question multiple-choice quiz, generated by ChatGPT and reviewed by our expert panel. These quizzes serve as a reinforcement tool, helping participants solidify their understanding of the key concepts covered in each module. Immediate feedback is provided for each question, with detailed explanations to clarify any misunderstandings and further support the learning process.

This comprehensive design, grounded in theory and informed by direct user feedback, ensures that the educational materials are not only accessible but also actionable, empowering patients with CRC and caregivers to take an active role in symptom management and care.

### Generating Accessible Multimedia Materials Using GenAI

**Figure 5.**
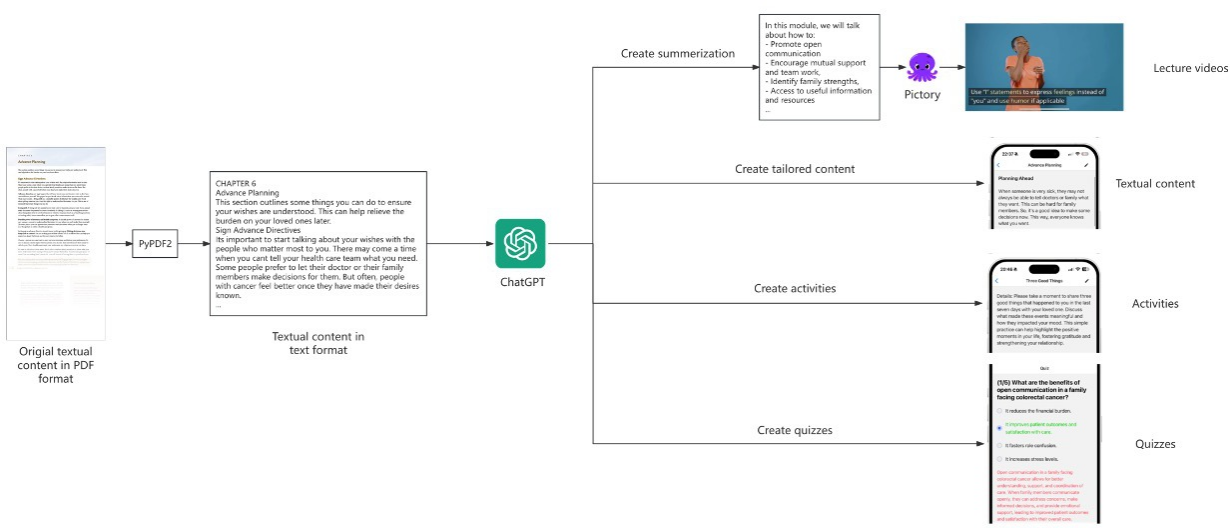
The framework was designed to leverage GenAI in creating multimedia content. First, the original PDF materials are converted into text using the Python package PyPDF2. The extracted text is then tailored by ChatGPT for 4 specific purposes: summarization of the topic, shortening the content to a lower reading level, creating practical activities, and generating quizzes. The topic summary is then processed by Pictory, which converts it into a video alongside a pleasant human reading voice. GenAI: generative artificial intelligence.

To enable the accessibility and engagement of educational materials, CRCWeb incorporates a framework for using GenAI to develop multimedia content, as shown in Figure 5. This framework begins by using the PyPDF2 [27] package to extract textual information from the original PDF documents. ChatGPT then generates concise summaries of the extracted text for each module. In line with design principle 4, these summaries are further processed by Pictory to create lecture videos tailored to individuals with limited vision and reading disabilities.

Additionally, the extracted text is processed by ChatGPT to produce various content formats tailored to the needs of disadvantaged populations with limited health literacy or low income. For instance, concise, low-reading-level text is generated to teach symptom management, while practical activities and quizzes are created to support hands-on learning and retention. These tailored formats, in adherence to design principles 2 and 3, are designed to make the educational content more accessible and actionable. The majority of the educational materials are created by GenAI-powered tools and subsequently reviewed by a team of oncology experts to ensure accuracy and relevance before being made available to patients and caregivers.

In the lecture section, we use ChatGPT to distill the core content of each module into a brief, cohesive summary that highlights the key topics. This summary is then processed by Pictory, a leading GenAI-powered video generation tool, which swiftly transforms text into engaging video content. These videos provide an alternative to text, allowing users to watch or listen to the material, reducing cognitive load and increasing engagement. As shown in Figure 5, Pictory creates relevant videos by incorporating the transcript and automatically highlighting key terms to reinforce understanding. The videos also include relaxing background music and a human voiceover to ensure a smooth, engaging viewing experience. For users who prefer text, we provide video transcripts that offer the same content in written form.

In the content section, CRCWeb presents text-based materials adapted from national guidelines for each topic. To ensure these materials are concise and easy to understand, as per design principle 3, ChatGPT is tasked with summarizing guideline documents into no more than 250 words with a Flesch-Kincaid Grade level of 6, making the content accessible to users of all literacy levels. A smart tagging system highlights key points using predefined tags, which are then reviewed and refined by our oncology experts to ensure clarity and relevance. Links to the original, full-length documents are included at the end of each section for users who wish to explore the source materials in more detail. Additionally, a text-to-speech feature powered by the React Native TTS package [28] provides an auditory option for users with vision impairments or reading difficulties. To prevent the tags from being read aloud, we use regular expressions to remove them from the audio transcript, ensuring a smooth listening experience.

In the activity section, we use ChatGPT to generate simple, practical activities that patients and caregivers can complete together. These activities are designed to reinforce the key concepts covered in the module and are structured to be easy to implement in daily routines. User feedback on these activities is collected through a rating system, which allows us to continuously improve their relevance and usability.

For the quiz section, ChatGPT generates 10 multiple-choice questions for each module, each accompanied by a detailed explanation of the correct answer. From these, we select the 5 most appropriate questions, refining them to match the challenge level needed to reinforce the material without overwhelming users. As shown in Figure 5, these quizzes provide immediate feedback with an explanation, helping participants reinforce the knowledge they learned from the module.

By integrating GenAI with expert review and accessibility features such as video, text, and audio options, CRCWeb ensures that its educational materials are engaging, user-friendly, and tailored to the needs of both patients with CRC and caregivers.

### Performance Evaluation of Large Language Models in Tailoring Educational Content

We compared 3 models from the GPT family (GPT-3.5 Turbo, GPT-4, and GPT-4 Turbo) to generate tailored content for disadvantaged patients with CRC and their caregivers with limited health literacy using predetermined prompts. The GPT-generated content was then evaluated by oncology experts and applied to CRCWeb.

To promote the accessibility and comprehension of educational materials for disadvantaged patients with CRC and their caregivers with limited health literacy, we structured prompts to have large language models (LLMs) produce content at a low reading level (Flesch-Kincaid Grade level of 6), maintain a word limit of 250, and provide Spanish and Chinese translations for each topic.

Our primary sources for content generation were education materials from the National Cancer Institute and the National Comprehensive Cancer Network guidelines [26]. We carefully selected 30 distinct topics that encompass a broad spectrum of content, including fatigue, depression, anxiety, pain, cognitive impairment, nutrition, and more. A subset of the topics was implemented in CRCWeb.

As reported in our prior work [29], the GPT family of models exhibited outstanding capability in tailoring educational materials for disadvantaged patients with CRC and their caregivers with limited health literacy or low income, only with deviations from the designated reading level.

## Pilot Study

We conducted an 8-week single-arm pre-post pilot clinical trial among patients and their caregivers in 2 cancer clinics as our test users to guide the development of CRCWeb. Among all 40 enrolled participants (20 patients and their caregivers), 22 of them came from disadvantaged backgrounds (ie, income ≤250% Federal Poverty Level, Medicaid, and uninsured), while the remaining 18 were from nondisadvantaged backgrounds, including individuals with higher incomes and adequate insurance coverage. The participants were instructed to download and navigate through CRCWeb by a clinical coordinator.

### Participant Satisfaction With CRCWeb

The average satisfaction score was 3.980 out of 5 (1=strongly disagree, 2=disagree, 3=partially agree, 4=agree, and 5=strongly agree), with minimal variation (0.004), indicating that participants found the platform helpful on average.

When comparing satisfaction levels between the nondisadvantaged and disadvantaged groups, the average scores were nearly identical: 3.985 for the nondisadvantaged group and 3.971 for the disadvantaged group, resulting in a minimal difference of 0.014.

A nonparametric Two One-Sided Test using the Mann-Whitney *U* test was applied. The results, presented in Table S3 in Multimedia Appendix 1, revealed that for Questions 1 and 2, which assessed overall satisfaction and perceived helpfulness of CRCWeb, the distributions between the 2 groups are equivalent (both *P*<.05). For Questions 3 through 6—addressing the relevance of content, clarity, understanding of personal situations, and skills for symptom management—both Test 1 and Test 2 were also significant (*P*<.05). This indicates that both groups rated CRCWeb similarly on these dimensions, with no notable differences in how they perceived the app’s relevance, clarity, or utility in managing symptoms. This demonstrates strong equivalence in the experiences of both groups in these areas. Finally, for Question 7, which focused on whether CRCWeb provided practical suggestions for everyday life, only Test 2 was significant (*P*=.001), while Test 1 was not (*P*=.05). This suggests that the disadvantaged group seems to have found CRCWeb more useful for providing practical, everyday suggestions compared to the nondisadvantaged group. This observation further reinforces the usability of CRCWeb for disadvantaged populations, as they perceived the platform to provide more practical support than their nondisadvantaged counterparts.

### User Engagement and Login Frequency

To assess user engagement, we tracked attendance records, adherence rate, and login frequency across the study period. A total of 40 participants were enrolled in the intervention and the retention rate was 75%. Among the 40 participants, 87.5% completed all 3 modules and logged into CRCWeb at least 3 times. There was a clear difference in engagement between the disadvantaged and nondisadvantaged groups. The disadvantaged group logged in 209 times in total, compared to 83 logins from the nondisadvantaged group, making the disadvantaged group 2.52 times more engaged. A Mann-Whitney *U* test was performed to verify whether this difference was statistically significant, and the results confirmed that the disadvantaged group had significantly higher login frequencies (*P*=.047).

## Lessons Learned and Future Directions

This viewpoint presents that by transforming textual data into multimedia components and tailoring educational content to the needs of low-health-literacy populations, CRCWeb addressed the significant health disparities that exist for disadvantaged groups with limited health literacy or low income [30]. Our assessment suggests that CRCWeb significantly enhanced user engagement for disadvantaged groups with limited health literacy or low income and achieved high levels of user satisfaction. This result is particularly noteworthy, as prior research has often highlighted lower engagement among disadvantaged populations in digital health interventions [31]. While our results demonstrated CRCWeb’s effectiveness in delivering accessible educational content, several limitations should be addressed in future directions.

First, language translations were not included in this pilot study as we only recruited English-speaking participants. Prior research indicates that LLM performance varies by language, performing better in high-resource languages like German, French, and Spanish, but less effectively in lower-resource languages such as Kannada and Occitan [32,33]. To make CRCWeb more inclusive for non-English speakers, future work will evaluate auto-translation features and incorporate LLM-based translation for additional languages. This will allow CRCWeb to serve a broader, multilingual population.

Second, while commercial GenAI-powered tools such as ChatGPT and Pictory help streamline content creation, they come with risks such as potential pricing increases and service suspensions. To mitigate these risks, future work will explore developing our own GenAI models based on open-source frameworks [34-36]. This will give CRCWeb greater control over its content generation processes and ensure long-term scalability and cost-effectiveness.

Finally, in terms of user engagement, while login frequency is an important metric, it does not fully capture the complexity of user interactions with CRCWeb. To improve measurement accuracy, future iterations will include native apps for each platform, enabling more detailed tracking of user behaviors such as screen time and in-app navigation. Although this approach requires greater development effort, it will provide more inclusive metrics of user engagement.

## Conclusions

Improving the accessibility of educational content on symptom management is essential to empowering patients with CRC and their caregivers, enabling them to more effectively manage symptoms throughout treatment. This is particularly vital for disadvantaged populations with limited health literacy or low income, who often lack access to national guidelines or frequent hospital-based care. CRCWeb addresses this challenge by leveraging GenAI-powered tools to transform overwhelming health care guidance into accessible multimedia formats, specifically tailored for patients with CRC and their caregivers. With features like low-reading-level text, engaging videos, and user-friendly navigation, CRCWeb ensures that patients and caregivers can better understand and manage their symptoms. Designed with a stakeholder-centered approach, the platform prioritizes the needs of its users, making it a valuable tool for improving health outcomes. Moreover, the scalable design of CRCWeb demonstrates its potential to be adapted for broader disadvantaged populations with limited health literacy or low income, extending its impact beyond patients with CRC and caregivers to enhance health care accessibility for diverse groups.

## Supplementary material

10.2196/68516 Multimedia Appendix 1 Supplementary material.

## Abbreviations

CRC: colorectal cancer
GenAI: generative artificial intelligence
LLM: large language model
REDCap: Research Electronic Data Capture
