# Supplementary material for: Leveraging Artificial Intelligence for Digital Symptom Management in Oncology: The Development of CRCWeb
Source: JMIR Cancer. 2025 Jun 16;11:e68516. doi: 10.2196/68516 (PMC12185034; doi:10.2196/68516)
Supplement: Multimedia Appendix 1 [file cancer-v11-e68516-s001.docx]

Table S1. Inclusion and exclusion criteria for recruiting patients and caregivers.

|  | **Patients** | **Caregivers** |
| --- | --- | --- |
| Inclusion criteria | Age ≥18 years | |
|  | Read and understand English | |
|  | Diagnosed with colon or rectum cancer, including stage I-IV |  |
|  | Receiving active chemotherapy +/- other treatment (e.g., surgery, radiotherapy, target therapy, immunotherapy) |  |
|  | Self-reported at least two psychoneurological symptoms (i.e., fatigue, depression, anxiety, sleep disturbance, and pain) in the past month measured by Common Terminology Criteria for Adverse Events (CTCAE) |  |
|  | Identified primary caregiver (family members or significant others identified by the patients as their primary source of emotional and physical support) |  |
| Exclusion criteria | Have unstable diseases (e.g., bipolar disorder, schizophrenia, cognitive impairment) | |
|  | Eastern Cooperative Oncology Group (ECOG) Performance Status > 2 | |
|  | Life expectancy < 6 months | |

Table S2. Questions used in the post-intervention satisfaction survey. Questions 1 and 2 were designed to gather feedback on the overall feasibility of the app, while questions 3 to 7 focused on assessing whether the educational materials were clear, comprehensible, and practical. Users rated each question on a scale of 1 to 5, with 1 representing "strongly disagree," 2 as "disagree," 3 as "partially agree," 4 as "agree," and 5 as "strongly agree."

| **Post-Intervention Satisfaction Survey** | |
| --- | --- |
| 1 | Overall, I'm satisfied with CRCWeb. |
| 2 | Overall, CRCWeb was helpful for me. |
| 3 | CRCWeb covered issues that were relevant to me. |
| 4 | The content of CRCWeb was clear and comprehensible. |
| 5 | CRCWeb helped me gain a better understanding of my situation. |
| 6 | CRCWeb provided me with skills to manage symptoms. |
| 7 | CRCWeb provided me with practical suggestions for everyday life. |

Table S3. Non-parametric TOST results comparing the non-disadvantaged and disadvantaged groups across 7 perspectives based on the post-intervention satisfaction survey assessing CRCWeb. Test 1 examines whether the disadvantaged group rated CRCWeb significantly lower than the non-disadvantaged group, while Test 2 checks if the non-disadvantaged group rated it significantly higher. Equivalence between the groups is concluded if both tests are significant (*P* < .05).

| **Perspective** | **P-value: Test 1** | **P-value: Test 2** |
| --- | --- | --- |
| 1. Overall satisfaction with CRCWeb | .000 | .001 |
| 2. Helpfulness of CRCWeb | .000 | .007 |
| 3. Relevance of issues covered | .000 | .007 |
| 4. Clarity of content | .001 | .001 |
| 5. Understanding of the situation | .000 | .004 |
| 6. Symptom management skills | .002 | .003 |
| 7. Practical suggestions for everyday life | .055 | .001 |
| 8. Average score | .000 | .003 |
